# Long-Term Follow-Up of Open Gustilo-Anderson IIIB Fractures Treated With an Adjuvant Local Antibiotic Hydroxyapatite Bio-Composite

**DOI:** 10.7759/cureus.39103

**Published:** 2023-05-16

**Authors:** Joshua A Henry, Almigdad Ali, Ibrahim H Elkhidir, Adam Reid, Jason Wong, Anand Pillai

**Affiliations:** 1 Department of Trauma and Orthopaedics, Wythenshawe Hospital, Manchester Foundation Trust, Manchester, GBR; 2 Department of Medicine, University of Khartoum, Khartoum, SDN; 3 Department of Plastic Surgery, Wythenshawe Hospital, Manchester Foundation Trust, Manchester, GBR

**Keywords:** metalwork infection, gustilo anderson iiib, open fractures, local antibiotics, limb salvage, cerament g, adjuvant antibiotic therapy

## Abstract

Background

Open fractures associated with significant tissue loss are complex and present challenges in management; they are associated with poor outcomes such as infection, non-union or amputation. This study aimed to evaluate outcomes of using an adjuvant local antibiotic hydroxyapatite bio-composite in the management of open Gustilo-Anderson IIIB fractures with up to eight years of follow-up.

Methods

This was a retrospective study. A total of 81 patients with Gustilo-Anderson IIIB fractures treated with “fix and flap” limb reconstruction with adjuvant local antibiotic therapy using a bio-composite carrier were reviewed.

Results

The mean follow-up time for all the patients, at the time of data collection, was 55.8 months. Union was achieved in 96% with a limb salvage rate of 96.3% and a deep infection rate of 3.7%.

Conclusion

The use of local antibiotic therapy, together with a combined orthoplastic “fix and flap” approach for Gustilo-Anderson IIIB open fractures, was found to be associated with a very low rate of metalwork infection and high union and limb salvage rates. Future studies should include some functional and quality of life outcome measures to see the efficacy of this method.

## Introduction

Open fracture management is complex, with the aim of preserving function while also preventing soft tissue or bone infections [1,2]. Gustilo-Anderson (GA) IIIB fractures are defined as open fractures with extensive tissue damage and significant periosteal stripping that do not have adequate soft tissue coverage [1]. Due to the amount of soft tissue damage and bone exposure associated with GA IIIB fractures, they are associated with higher infection rates than those classified as GA I to IIIA [3,4].

The current surgical management of these injuries involves early wound debridement, fracture stabilisation, and soft tissue coverage alongside early and perioperative systemic antibiotic therapy [2]. More recent developments have seen the introduction of local adjuvant antibiotic therapy with the aim of increasing the concentration of antibiotic at the fracture site and this has been shown to significantly reduce rate of infection compared with systemic antibiotics alone [5]. The use of these, however, has been slow to be taken up, perhaps due to the lack of a reliable delivery system.

Cerament G is an injectable biphasic synthetic bone filler consisting of hydroxyapatite, calcium phosphate and gentamicin. Its constitution enables it to resorb slowly over months meaning that the gentamicin stays locally in situ with a concentration greater than the minimum inhibitory concentration (MIC) for at least 28 days and is able integrate with bone and has good radiological remodelling potential [6,7]. The aim of this study was to assess the long-term outcomes for patients with Gustilo-Anderson IIIB open fractures treated with adjuvant local antibiotic therapy, specifically limb salvage, union, and reinfection rates.

## Materials and methods

This was a retrospective cohort study. The study included all patients with GA IIIB open fractures that were treated by the Manchester Orthoplastic Group between June 2013 and April 2021 with a fix and flap approach. The patients in this study were a combination of both tertiary referrals from regional “spoke” hospitals as well as patients who presented directly through the local emergency department. We excluded all patients with open fractures that had adequate tissue coverage not requiring a flap (i.e., non-IIIB fractures).

At presentation, all patients were treated in line with British Orthopaedic Association Standards for Trauma and Orthopaedics (BOAST) guidelines for open fractures [8]. Systemic antibiotics were prescribed in line with local microbiology guidance and hospital protocols until definitive wound closure was achieved. Where patients were referred from spoke hospitals, the initial debridement and wound care was conducted at the presenting hospital. Patients were transferred to the Manchester Orthoplastic Group once they were ready for definitive fixation and skin coverage. All patients underwent definitive skeletal stabilisation followed by immediate soft tissue coverage. Cerament G was mixed according to the manufacturer’s instructions and injected locally within the wound. Soft tissue and bone samples were taken at the time of definitive surgery and analysed for extended culture and sensitivities.

Patients were followed up at regular intervals as per clinical need. Data collected included basic patient demographics (age, gender, site and mechanism of injury), peri-operative data including first debridement after injury, time of definitive “fix and flap” session, type of skeletal stabilisation and of soft tissue cover. We used clinical letters, electronic patient records, radiology investigations and primary care portal notes to acquire the data for this study. The primary outcomes of this study were infection rate (both superficial and deep), union rate and limb salvage rate. Secondary outcomes included time to union, reoperation rate and complications other than infection. All analysis was completed using Microsoft Excel software (Microsoft, Redmond, WA). Confidence intervals (CIs) were calculated to 95% probability.

## Results

A total of 81 patients met the inclusion criteria for analysis in this study with a mean follow-up duration, at the time of data collection, of 55.8 months (range 11-101 months). Patients were from a wide age range, from 9 to 91 years, with a mean age of 39.9 years; 18 (22%) were female and 63 male (78%). There were a variety of mechanisms of injury with road traffic accidents being the most common (55.6%). Sites of injury included tibial diaphyseal in 63.75% and distal tibia and ankle in 17.5% cases (Table 1).

**Table 1:** Baseline patient demographics and fracture location

Patient demographics (n=81)		
Age		
Mean	39.5	
Range	9-88	
	n	%
Gender		
Male	64	79%
Female	17	21%
Smoking		
Yes	45	56%
No	36	44%
Fracture site		
Tibia + fibula (diaphyseal)	38	42%
Tibia (diaphyseal) only	14	16%
Tibia + fibula (distal intraarticular)	9	10%
Ankle	14	16%
Foot	11	12%
Femur	3	3%
Hand	1	1%
Patients with multiple fractures	8	10%

All patients attended theatre for initial debridement within 48 hours of presentation (mean 11.21 hours, range 3-40 hours, CI 9.7-12.7). Definitive procedures were performed within seven days of injury for 41 patients (mean 9.52 days, range 1-53 days, CI 7.8-11.2 days). Delays in definitive fixation were a result of varying factors including optimisation of medical comorbidities, availability of an appropriate orthoplastic theatre list or a delay in transfer from a referring hospital.

Skeletal fixation was achieved via multiple methods (Table 2). The method of soft tissue coverage was dependent on the severity of soft tissue loss, site of injury and availability of healthy soft tissue for transplant. The most common soft tissue coverage was a free anterolateral thigh (ALT) flap (46.9%).

**Table 2:** Breakdown of operations (both the bony and soft tissue procedures) performed on the study population ORIF, open reduction and internal fixation

Surgical procedure (n=81)	n		%
Skeletal stabilisation			
ORIF		25	30.9%
Circular frame		19	23.5%
Combination ORIF + circular frame		4	4.9%
External fixator		1	1.2%
Combination external fixator + ORIF		11	13.6%
Intramedullary nail		16	19.8%
K-wires		3	3.7%
Other		2	2.5%
Soft tissue coverage			
Anterolateral thigh free flap		38	46.9%
Locoregional (pedicled) flap		29	35.8%
Skin graft		8	9.9%
Latissimus dorsi free flap		3	3.7%
Other		3	3.7%

Amputation was required for three patients, resulting in a limb salvage rate of 96.3%. Two of these patients had amputation within two weeks after surgery due to flap failure and extensive tissue loss; the third patient had their amputation at eight weeks following definitive surgery due to flap congestion and necrosis. These patients were excluded from calculation of union rates.

Bony union was achieved in 75 patients (96%) with a mean time to union of 11.6 months (range 3-48 months, CI 9.5-13.7 months). Primary bony union without secondary intervention was achieved in 64 (82%). There were 18 cases of delayed union (25%) of which eight required further intervention. Four patients had the Exogen Ultrasound Bone Healing System (Bioventus, Durham, NC) applied, and four had bone marrow aspirate concentrate (BMAC) injected into the fracture site, following which union was achieved in all patients.

Non-union, as defined by no radiological evidence of bony healing at 12 months following fixation, was documented for three patients (5.6%). The index skeletal stabilisation procedure for these patients was circular frames. These were all revised to internal fixation along with autologous cancellous bone graft. At the time of revision operation, deep tissue samples did not isolate any infection. All three patients went on to achieve bony union by 30 weeks after revision fixation.

A total of 31 patients (38%) in this cohort experienced no complications after definitive surgery. The most common complication was superficial wound infection, occurring in 36 patients (44.4%) at a mean time of three months after definitive surgery (range 0-16). There were three cases of infected metalwork (3.7%). There was failure of the flap in five patients (6%) and wound dehiscence in one patient.

## Discussion

GA IIIB fractures are potentially devastating injuries that were once associated with very high infection rates of up to 52% and non-union rates of 50% [9]. More recent advancements in the management of these patients have led to an improvement in these figures; however, there are still reports of infection rates up to 15% and non-union of up to 29% leading to poor functional outcomes [10-12].

Aljawadi et al. published the mean 22-month follow-up data for patients who underwent “fix and flap” fixation of IIIB fractures with the application of a hydroxyapatite bio-composite containing gentamicin in the Manchester Orthoplastic Unit [13]. They reported a limb salvage rate of 96% and a primary union rate of 88%. Infection was reported in 17 patients (21%); 15 of these infections were superficial infections treated with systemic antibiotics and the remaining two were classified as deep infection, one relating to the flap and the other to the metalwork. Both patients required revision surgery. There were 15 returns to theatre: six for delayed union, three for non-union, five for flap-related complications and one removal of metalwork. One criticism of that paper was the relative short follow-up period of just 22 months. This paper addresses those concerns.

This article now represents what we believe to be the largest published cohort of GA IIIB patients with a mean follow-up of 55 months. Our study shows that the “fix and flap” method of skeletal stabilisation and soft tissue coverage with adjuvant local antibiotic therapy has union rates and limb salvage rates similar or better than other published studies, as described in Table 3 [2,14,15].

**Table 3:** Comparison of Gustilo-Anderson IIIB fracture outcomes from the literature

	Number of patients	Follow-up (months)	Deep infection rate (%)	Union rate (%)	Limb salvage rate (%)
Gopal et al. [2]	79	-	9.5	66	95
Naique et al. [15]	73	14	8.5	51	93
Mathews et al. [16]	74	12	14		92
Wordsworth et al. [14]	66	40	1.5	89.4	94
Aljawadi et al. [13]	80	22	2.5	88	96
This study	81	55	3.7	96	96.3

**Figure 1:**
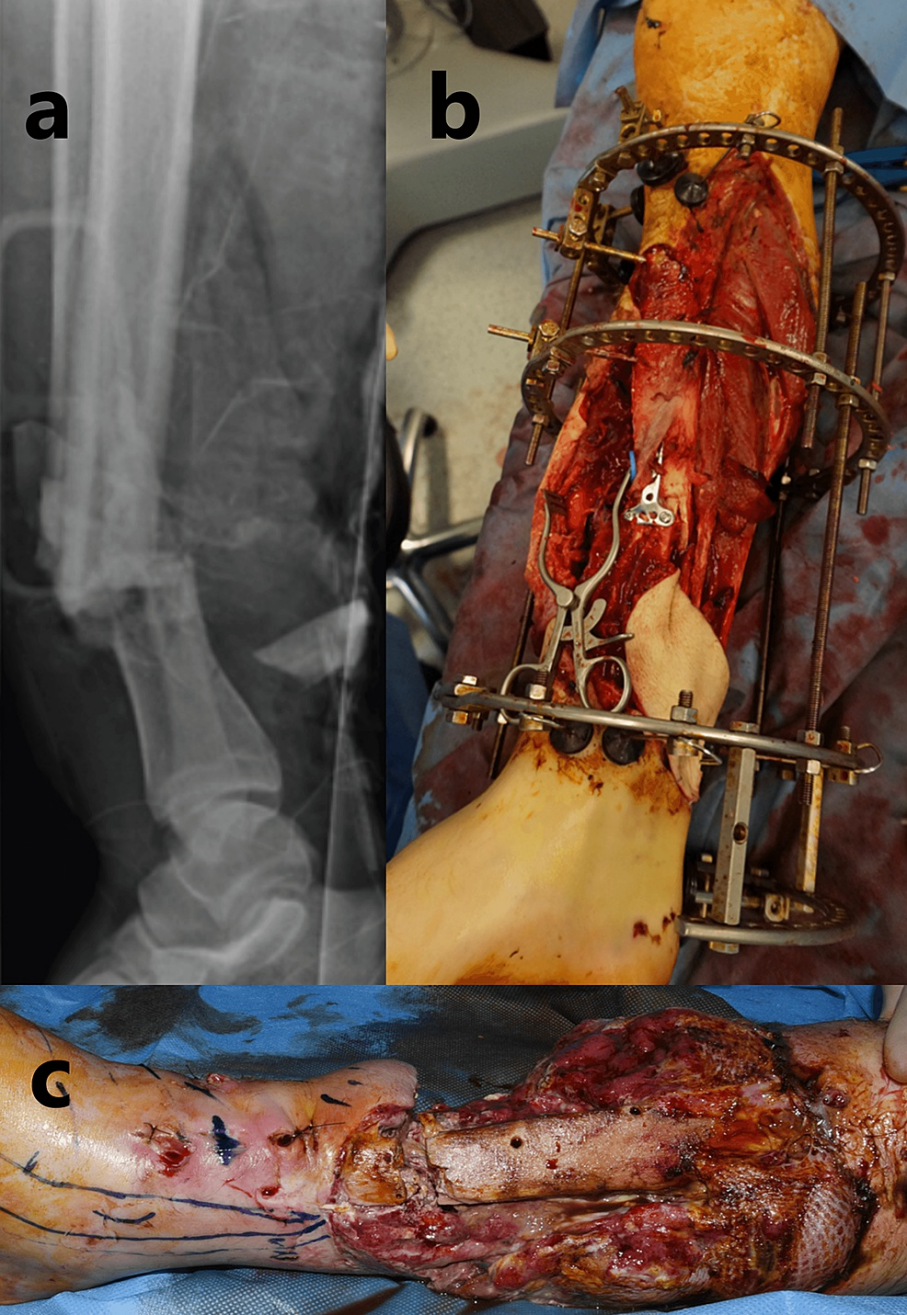
Unsuccessful treatment of the open fracture with fix and flap resulting in non-union: (a) radiograph of the lower limb open fracture, (b) intraoperative clinical photograph at the time of definitive fixation showing skin loss and method of fixation with frame and internal fixation, (c) intraoperative clinical photograph showing atrophic non-union with a large fracture gap and flap failure

The superficial infection rate increased from 21% at 22 months to 44% in this most recent study. This is possibly due to the implementation of new IT systems that enabled easier access to primary healthcare records since the original study. There were two further cases of metalwork infection, increasing the rate from 1.25% to 3.75%. *Staphylococcus aureus* was the responsible organism for two of these infections, one of which was sensitive to gentamicin. The third patient had *Klebsiella oxytoca* and *Serratia liquefaciens* isolated in the first 12 months following definitive fixation. This was treated as a superficial infection with surgical washout and debridement followed by a prolonged course of antibiotics. This patient then went on to develop osteomyelitis 52 months after definitive fixation with methicillin-resistant *S. aureus* (MRSA) and methicillin-susceptible *S. aureus* (MSSA) being isolated at the time of metalwork removal. All three patients had tibia and fibula fractures; two patients had open reduction and internal fixation and the third had an intramedullary nail for definitive skeletal stabilisation. All three patients had local advancement flap coverage for their soft tissue injuries. The metalwork was removed at 6 months, 16 months and 52 months for the three patients. Infection was irradicated in all three patients. Figure 1 shows clinical photography and radiograph images for one of these patients. Although this study shows a higher superficial infection rate, there remains a very low (3.75%) rate of deep infection. This is much improved compared to that described by Mathews et al. [16], and equivocal to other studies with smaller cohorts and short-term follow up [2,14,15].

**Figure 2:**
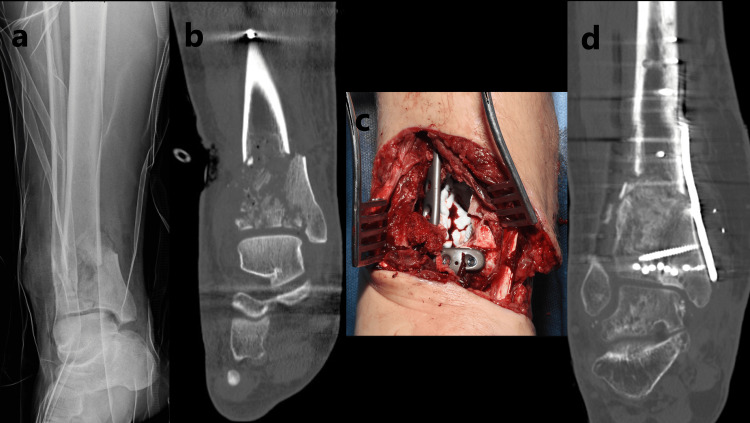
Successful treatment of the open fracture with an adjuvant local anaesthetic: (a) plain radiograph of the open distal tibia fracture, (b) coronal CT image of the distal tibia fracture, (c) intraoperative clinical photograph showing internal fixation with bio-composite cement underneath the plate in the bone void, (d) postoperative coronal CT image showing ossification of the bone void and signs of union

It is widely accepted that antibiotics play a key role in the prevention of infection in patients with open fractures; however, the severe nature of soft tissue disruption associated with GA IIIB fractures leads to the devascularisation of the area and, as a result, poor perfusion [3,4]. Tissue penetration of the antibiotic may therefore be reduced, resulting in a lower concentration of the antibiotic at the fracture site [17]. In addition to this, open fracture patients will mostly have the added requirement of skeletal fixation with orthopaedic implants. Colonisation and adherence of bacteria can form a biofilm on the surface of these implants that can shield the microorganisms from both antibiotics and the host's immune system leading to antibiotic resistance [18]. The minimum biofilm eradication concentration (MBEC) of antibiotics can be up to 100-1000 times higher than the MIC or minimum bactericidal concentration (MBC) and can often not be achieved with systemic antibiotics, especially in areas of poor perfusion [19]. The use of adjuvant local antibiotic solutions such as Cerament G has been shown to elute a local concentration of gentamicin greater than 250 times its MIC for the first seven days and more than 10 times the MIC at 28 days after use [20]. Morgenstern et al. conducted a systematic review and meta-analysis to assess the impact of local antibiotic therapies on open fractures [21]. They showed a reduction in the infection rate of all open fractures from 16% to 4% with the largest impact on GA III fractures: reducing the infection rate from 27% to 7%. Craig et al. reported similar outcomes in their review, with a reduction of infection rates from 31% to 9% with the addition of adjuvant local antibiotics for GA IIIB and C fractures [22]. The results of our series show comparable results with a deep infection rate of just 3.7%. Figure 2 shows a patient in this series who was successfully treated via this method.

The main limitation of this study is that due to the nature of referrals from spoke hospitals, patients who may have presented back to their local units with late complications may have been missed. To counteract this, we looked at primary care records and radiography of all patients to try to capture any patients that this may have applied to. Another limitation is the retrospective nature of our data collection. It may be useful to include some functional and quality of life outcome measures in future research looking at the efficacy of this method for treating open fractures.

## Conclusions

The use of the hydroxyapatite bio-composite loaded with gentamicin for local delivery into the tissue is an emerging and readily available treatment adjunct for open fractures with significant tissue loss. Our study shows that its use is associated with a 3.75% rate of deep infection, a high rate of union (96%) and a limb salvage rate of up to 96% at up to eight years of follow-up. This study of 81 patients represents one of the largest cohorts with a significant length of follow-up, and shows that a concurrent definitive skeletal stabilisation with soft tissue coverage and adjunctive local antibiotic administration can lead to excellent long-term results.
